# Influence of Adhesion Promoter Primers on Polymerization Kinetics and Long-term Bond Strength of Composite Cements to Zirconia

**DOI:** 10.3290/j.jad.b3146833

**Published:** 2022-06-20

**Authors:** Bruna Marin Fronza, Mayara dos Santos Noronha, Richard Bengt Price, Vanessa Gallego Arias Pecorari, Marcelo Giannini

**Affiliations:** a Postdoctoral Fellow, Department of Biomaterials and Oral Biology, University of São Paulo, SP, Brazil. Experimental design, performed the experiments, and wrote the manuscript.; b PhD Student, Department of Restorative Dentistry, Piracicaba Dental School, University of Campinas, Piracicaba, SP, Brazil. Performed the experiments.; c Professor, Department of Clinical Dental Sciences, Faculty of Dentistry, Dalhousie University, Halifax, NS, Canada. Experimental design, reviewed the manuscript.; d Professor, Department of Biostatistics, Paulista University, São Paulo, SP, Brazil. Performed statistical analysis.; e Professor, Department of Restorative Dentistry, Piracicaba Dental School, University of Campinas, Piracicaba, SP, Brazil. Experimental design, reviewed the manuscript.

**Keywords:** adhesion, ceramics, indirect restoration, primer, composite cement, surface treatments, zirconia.

## Abstract

**Purpose:**

To investigate the influence of primers on polymerization kinetics of resin-based luting and its effect on the microhardness and bond strength to zirconia.

**Materials and Methods:**

Panavia V5 (PV; Kuraray Noritake) with Tooth Primer (TP_primer; Kuraray Noritake_) or Clearfil Ceramic Primer (CP_primer; Kuraray Noritake_), and RelyX Ultimate (RU; 3M Oral Care) with Scotchbond Universal (SU_adhesive; 3M Oral Care_) were evaluated. Polymerization kinetics of luting materials with or without primers (TP_primer_ or SU_adhesive_) were evaluated using Fourier transform near-infrared (FT-NIR) spectroscopy in self- and dual-curing modes (n = 5). Microhardness of luting materials was evaluated after 1, 12, and 24 h (n = 5). Shear bond strengths to zirconia ceramics (Katana Zirconia, Kuraray Noritake; and Lava Esthetic, 3M Oral Care) after 24 h and 1 year (n = 8) were assessed to determine the effect of the following surface treatments: no treatment, non-thermal atmospheric plasma, primer (CP_primer_ or SU_adhesive_), and the combination of plasma + primers. Statistical analyses were performed at a 5% significance level.

**Results:**

PV achieved a significantly higher degree of conversion (DC) when TP_primer_ was used, while there was no increase in conversion for RU combined with SU_adhesive_. Light activation significantly improved polymerization, which also produced greater microhardness. CP_primer_ and SU_adhesive_ significantly improved immediate bond strength to zirconia ceramics. However, after 1 year, only SU_adhesive_ with RU was able to maintain the bond strength. Plasma surface treatment did not improve bonding to zirconia.

**Conclusion:**

The use of primers improved the DC for PV only. Light curing produced higher conversion and microhardness for both resin-based luting materials. Bond strength to zirconia was improved when primers were used. However, only RU demonstrated reliable long-term adhesion to zirconia.

When using glass ceramics, etching the surface with hydrofluoric acid followed by applying a silane coupling agent provides reliable adhesion.^35^ Metal-oxide ceramics such as zirconia cannot be acid etched; careful air-particle abrasion is recommended instead.^4,27,34^ Furthermore, because this ceramic does not contain silica, the silane cannot chemically bond to zirconia.^15^ The use of acidic monomers capable of establishing a chemical bond to oxides, such as 10-methacryloyloxydecyl dihydrogen phosphate (10-MDP) incorporated into luting materials, primers, and adhesives, have been recommended to enhance bond strength to zirconia.^15,34,36,38^ In fact, clinical studies demonstrate good clinical survival of resin-bonded zirconia partial dentures using primers and/or luting materials containing these acidic monomers after three and six years.^29,30^ However, debonding of this material is also often reported in clinical studies, especially when the preparation design has little or no mechanical retention.^4,27^ Nevertheless, because zirconia ceramic has high flexural strength, high fracture toughness, is cost-effective, and appears more tooth-like than metal, researchers and industry are pursuing optimal bonding protocols for this material.^39^ Alternative surface treatment methods, such as nonthermal atmospheric plasma have also been suggested, because plasma can increase the surface energy and wettability,^10,14^ factors that favor adhesion.^37^

A luting material’s properties are, in turn, related to its polymerization. The polymerization reaction can be chemically initiated when the component pastes are mixed; alternatively, photoinitiators in the material can be activated by light (light curing), or a combination of both mechanisms in a dual-curing system.^7,19^ Previous studies have reported a higher degree of conversion (DC) and consequently improved physicochemical properties and adhesion to substrates when the resin-based luting materials were dual-cured as compared to self-cured.^13,18,28,33^ However, light attenuates considerably as it passes through indirect materials. For example, it was reported that 0.5 mm of feldspathic ceramic reduced the irradiance by 50%, and 0.5 mm of zirconia reduced the irradiance by 67%.^23^ However, the amount of light attenuation differs according to materials’ microstructure, shade, and thickness. The greater the light attenuation, the lower the polymerization rate of the luting material,^11,12^ so that the DC becomes more dependent on chemical activation. Furthermore, if the chemical redox initiating system uses benzoyl peroxide as the oxidant and tertiary amines as reductant, the low pH of the primers or the acidic monomers in self-adhesive materials may interfere with polymerization. Some manufacturers use alternative systems, such as the combination of cumene hydroperoxide or sodium persulfate, associated with benzoyl thiourea or tert-butyl peroxide trimethylhexanoate, as the reducing agent.^9,19^ However, the entire composition is proprietary and is not fully disclosed by manufacturers. Instead, they often recommend matching their luting material with their specific primers or adhesives to overcome these chemical incompatibilities.

Thus, the objective of this study was to investigate whether the use of primers affects the polymerization kinetics of the resin-based luting material in the self- and dual-curing modes, its microhardness, and the 24-h and 1-year bond strength to zirconia ceramics, associated with or without non-thermal atmospheric plasma. The hypotheses tested were: (1) the use of primers increases the DC and polymerization rate of the tested luting materials; (2) light curing increases the microhardness of the dual-curing resin-based luting materials compared to self-curing only; (3) treating the zirconia surface with primers increases the bond strength to the tested luting materials; and (4) treating the zirconia surface with non-thermal atmospheric plasma improves bond strength to the tested resin-based luting materials.

## MATERIALS AND METHODS

Two resin-based luting materials combined with their primer/adhesive from the same manufacturer were tested: Panavia V5 (PV; Kuraray Noritake; Tokyo, Japan) used with either Panavia V5 Tooth Primer (TP_primer_), or Clearfil Ceramic Primer (CP_prime_); and RelyX Ultimate (RU; 3M Oral Care; St Paul, MN, USA) used with Scotchbond Universal Adhesive (SU_adhesive_). The material information and chemical composition provided by the manufacturers are reported in Table 1. Materials were evaluated in both their self- and dual-curing (ie, with light activation) modes.

**Table 1:** Materials evaluated and respective manufacturers’ information and application modes.

Classification	Material	Manufacturer	Info	Composition*
Resin-based luting materials	Panavia V5 (PV)	Kuraray Noritake (Tokyo, Japan)	Lot 3M0018 Shade A2	Bis-GMA, TEG-DMA, hydrophobic aromatic dimethacrylate, hydrophilic aliphatic dimethacrylate, camphorquinone, initiators, accelerators, and pigments Fillers: silanated barium glass, silanated fluoroaluminosilicate glass, colloidal silica, surface treated aluminum oxide
	RelyX Ultimate (RU)	3M Oral Care (St Paul, MN, USA)	Lot 3391306 Shade A2	TEG-DMA, 2-propenoic acid, 2-methyl-1,1’-[1-(hydroxymethyl)-1,2-ethanediyl]ester, reaction products with 2-hydroxy-1,3-propanediyl dimethacrylate and phosphorus oxide, sodium persulfate, tert-butyl peroxy-3,5,5-trimethylhexanoate, acetate monohydrate. Fillers: silane-treated glass, silane-treated silica, oxide glass chemicals
Primers and adhesive	Panavia V5 Tooth Primer (TP_primer_)	Kuraray Noritake	Lot 3D0050	HEMA, 10-MDP, hydrophilic aliphatic dimethacrylate, accelerators, water
	Clearfil Ceramic Primer (CP_primer_)	Kuraray Noritake	Lot 15033	Ethanol, MPS, 10-MDP
	Scotchbond Universal Adhesive (SU_adhesive_)	3M Oral Care	Lot 3757B12	10-MDP, bis-GMA, HEMA, 2-propenoic acid, 2-methyl-, reaction products with 1,10-decanediol and phosphorous oxide, ethanol, water, copolymer of acrylic, and itaconic acid, camphorquinone, ethyl 4-dimethylaminobenzoate
Ceramics	Katana Zirconia	Kuraray Noritake	Lot BNAHZ Shade KT10	Zirconium oxide, yttrium oxide, pigments
	Lava Esthetic	3M Oral Care	Lot 190676 Shade A2	Zirconia ceramic

*Information supplied by the manufacturer. Abbreviations: bis-GMA: bisphenol-A diglycidyl ether dimethacrylate; HEMA: 2-hydroxyethyl methacrylate; 10-MDP: 10-methacryloyloxydecyl dihydrogen phosphate; MPS: 3-trimethoxysilylpropyl methacrylate; TEG-DMA: triethyleneglycol dimethacrylate.

### Polymerization Kinetics

The luting materials were mixed using their respective automix tips and were placed in a metal ring that was 12 mm in diameter and 0.5 mm thick between two glass slides. TP_primer_ and SU_adhesive_ primers were tested with their respective luting materials that had been applied on one side of the glass slides, following manufacturers’ instructions (primer application for 20 s followed by drying with mild air). This simulated the application of the primer to the tooth surface and the sandwiching of the luting material. The real-time polymerization kinetics of luting materials (n = 5) was monitored by Fourier transform near-infrared spectroscopy (FT-NIR, Tensor 27, Bruker; Billerica, MA, USA). The changes in the area of the methacrylate vinyl absorbance band centered at 6165 cm^-1^ were used to follow the polymerization reaction.^32^ Measurements were taken at a wavenumber resolution of 4 cm^-1^ with 4 scans per spectrum at a scanner velocity of 20 Hz. Data was collected continuously for 10 min immediately after mixing the luting material and was either allowed to self-cure or it was light activated. The Valo Cordless (Ultradent; South Jordan, UT, USA) light-curing unit was positioned 5 mm away from the specimens, which received 17.2 J/cm^2^ when exposed for 20 s at an irradiance of 860 mW/cm^2^ at this distance. These values were obtained using a spectroradiometer (USB4000, Ocean Insight; Largo, FL, USA). For each data point of the methacrylate vinyl absorbance peak area (total of 13,000 data points in 10 min) collected, the DC was calculated using the following formula:


$$\text{ Degree of conversion }=1-\left(\frac{\text{ peak area }}{\text{ peak area at data point }1}\right)$$

DC results were then plotted over time, and the maximum polymerization rate (PR_max_) was calculated as the first derivative of the conversion vs time curve. Data distribution was normal and homoscedastic according to Levene and Shapiro-Wilk tests, respectively, with the exception of degree of conversion data for RU, which was transformed by square root function (SigmaStat 3.5, Systat Software; San Jose, CA, USA) in order to fulfil the parameters of normality and homoscedasticity for a parametric analysis. After that, data were analyzed by one-way ANOVA separately for each luting material (SigmaStat). Tukey’s post-hoc tests were used to detect differences among the groups using a pre-set α of 0.05.

### Microhardness

Using the same conditions that were used in the polymerization kinetics experiment, disk-shaped specimens of luting materials (n = 5) were fabricated using a metal ring 12 mm in diameter and 1 mm thick between two glass slides. Materials were allowed to self-cure, or they were light activated immediately from the top after mixing and dispensing. Microhardness was measured using a microhardness tester (Mitutoyo HM123; Toronto, ON, Canada). The Vickers diamond indenter was applied with a static load of 50 grams for 10 s. Five indentations per specimen were made at the top surface (ie, the surface closest to the light curing unit for light-activated specimens) at 1, 12, and 24 h after mixing the luting material. Specimens were dried and stored in the dark at room temperature (23 ± 1°C) between measurements. Data distribution was normal and homoscedastic according to Levene and Shapiro-Wilk tests, respectively, with the exception of RU data, which was transformed by square root function to fulfil the parameters of normality and homoscedasticity for a parametric analysis. After that, data were subjected to repeated-measures two-way ANOVA (SigmaStat). Tukey’s post-hoc tests were performed to detect significant differences among groups using a pre-set α of 0.05.

### Shear Bond Strength (SBS)

Zirconia ceramic plates (10 mm long x 5 mm wide x 1 mm thick) of Katana Zirconia KT10 (Kuraray Noritake) and Lava Esthetic (3M Oral Care) were made and sintered according to manufacturers’ recommendations. Adhesive tape with two 4-mm-diameter holes was placed on each zirconia plate to define the same bonding area (12.57 mm^2^) for all the luting materials. The bonding areas were then lightly sandblasted with 50-μm Al_2_O_3_ (Danville Engineering; San Ramon, CA, USA) for 10 s (air pressure: 0.25 MPa; distance from the tip: 10 mm) and ultrasonically cleaned for 5 min.

Specimens were divided into 16 experimental groups (n = 8), according to zirconia type: Katana and Lava; luting materials: PV or RU; and surface treatment: no treatment, non-thermal atmospheric plasma, primer (CP_primer_ or SU_adhesive_), and the combination of plasma + primer. Plasma was applied to the zirconia surface for 30 s (argon gas with a flow rate of 5.0 l/min; nozzle positioned 10 mm from the surface in static mode).^21^ A thin coat of primer was applied with a microbrush and then air dried for 10 s using a gentle stream of oil-free air.

A device with a cylindrical Teflon mold (bonding clamp and bonding mold inserts, Ultradent) was used to build two cylinders (2.4 mm in diameter and 3 mm in height) of luting material over each treated zirconia plate. For each specimen, the luting material was inserted into the mold and light cured for 20 s with the Valo light-curing unit. Subsequently, the mold was removed, and the specimens were stored in deionized water at 37°C for either 24 h or 1 year before performing the SBS test. The deionized water was changed every month for the specimens that were stored for 1 year.

For the SBS test, the specimens were fixed with cyanoacrylate glue in a cylindrical acrylic-resin mold and attached to a shear testing device following the instructions provided in ISO/TS 11405.^31^ The tests were conducted with a universal testing machine (EZ Test, Shimazu; Kyoto, Japan). Shear load was applied to the adhesive interface at a crosshead speed of 1 mm/min until the bond failed. The maximum stress before fracture was recorded, and bond strengths were obtained by dividing the maximum load by the bonding area (MPa). The data were asymmetrically distributed. Thus, for comparison among factors, generalized linear models were adjusted according to a 2 x 8 x 2 factorial design. The analyses were performed by the PROC GENMOD procedure of the SAS 9.3 program (SAS Institute; Cary, NC, USA), adjusted in the Poisson distribution, and multiple comparisons were verified by the Wald test. A pre-set α of 0.05 was used for the statistical analyses.

The interfacial zones where the specimens failed in the SBS test were examined with an optical microscope at 100X original magnification (KH 8700; Hirox, Tokyo, Japan). The failure modes were classified either as adhesive (debonding between luting material and zirconia) or mixed (adhesive failure combined with cohesive failure within the resin-based luting material).

## RESULTS

**Fig 1:**
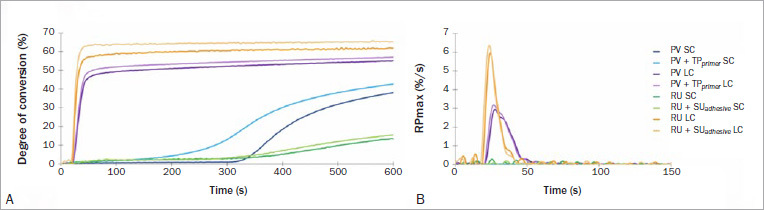
Polymerization kinetics for resin-based luting materials in self-curing (SC) and light-curing (LC) modes when used with their own adhesive and primer. (A) Real-time polymerization showed a slower increase in the degree of conversion and lower final conversion of both luting materials in the self-curing mode. (B) Note the maximum polymerization rate was greater when the luting materials were light cured.

The DC of the resin-based luting materials after 10 min and PR_max_ are reported in Table 2, and the polymerization kinetics profile of the conversion vs time and the polymerization rate are depicted in Fig 1. PV had a significantly higher degree of conversion when light activated and/or combined with TP_primer_, ie, the self-curing mode also had a statistically significantly greater degree of conversion with primer (F = 475.85, p < 0.001). The PR_max_ was significantly lower in the self-curing mode independent of primer/adhesive application, but the polymerization rate increased when the luting material was light cured (F = 1880.827, p < 0.001). In contrast, for RU, the use of SU_adhesive_ did not change the DC or PR_max_. Statistically significant differences were only found for the polymerization mode, where light curing significantly increased both the conversion and the polymerization rate (F = 561.834, p < 0.001).

**Table 2:** Mean (standard deviation) of degree of conversion (%) and maximum polymerization rate (%/s) for luting material at different conditions

	Degree of conversion (%)	PR_max_ (%/s)
Panavia V5 SC	38.1 (1.4) d	0.3 (0.1) c
Panavia V5 + TPprimer SC	42.7 (0.8) c	0.2 (0.1) c
Panavia V5 LC	55.2(0.5) b	2.9 (0.1) b
Panavia V5 + TPprimer LC	57.1 (0.7) a	3.2 (0.1) a
RelyX Ultimate SC	13.4 (2.2) b	0.4 (0.1) b
RelyX Ultimate + SUadhesive SC	15.5 (2.2) b	0.3 (0.1) b
RelyX Ultimate LC	63.3 (2.1) a	6.1 (0.8) a
RelyX Ultimate + SUadhesive LC	65.2 (1.7) a	6.7 (0.3) a

Within a column, means (n = 5) followed by the same letter are not statistically different (p > 0.05). The different luting materials (Panavia and RelyX Ultimate) were not statistically compared. LC: light curing; SC: self-curing; TP_primer_: Panavia V5 Tooth Primer; SU_adhesive_: Scotchbond Universal Adhesive.

The microhardness results are reported in Table 3. There was no statistically significant interaction between the factors “time” and “polymerization mode” (PV: p = 0.357; RU: p = 0.176). There was no significant influence of the post-polymerization time on the microhardness of the luting materials after 1 h (PV: F = 1.695, p = 0205; RU F = 3.917, p = 0.065). However, light curing significantly increased the microhardness for both PV (F = 310.983, p < 0.001) and RU (F = 5570.74, p < 0.001).

**Table 3:** Mean (standard deviation) of Vickers hardness (HV) for luting materials at different polymerization conditions and times

Time after polymerization (h)	Panavia V5		RelyX Ultimate	
	SC	LC	SC	LC
1	20.3 (1.5) Ab	36.2 (3.3) Aa	3.1 (0.7) Ab	56.1 (2.8) Aa
12	23.3 (1.4) Ab	36.6 (3.0) Aa	4.1 (0.9) Ab	56.5 (1.0) Aa
24	23.2 (1.4) Ab	36.3 (1.8) Aa	4.1 (0.5) Ab	56.7 (2.6) Aa

Means (n = 5) followed by the same letter (uppercase compares rows [time], lowercase compares columns [polymerization mode]) are not statistically different (p > 0.05). The different luting materials (Panavia and RelyX Ultimate) were not statistically compared. LC: light curing; SC: self-curing.

The SBS of the luting materials to both of the zirconia ceramics are reported in Table 4. Statistical analysis showed no difference for the “ceramic” factor (p = 0.7744), but there were differences for the “treatment” (p < 0.0001) and “time” (p < 0.0001) factors. In terms of interaction, significant differences in “treatment x time” (p < 0.0001) were found. For both ceramic materials, some pre-test sample failures occurred in the PV treated with plasma group and in the untreated group. These values were included as zero in the data tabulation and in the statistical analysis. For Katana Zirconia, after 24 h storage, the application of CP_primer_ or SU_adhesive_ with and without plasma yielded higher bond strengths. Using plasma alone produced similar results to CP _primer_ and plasma+SU_adhesive_, but only for RU luting material. When no surface treatment was done, ie, sandblasting only, RU demonstrated higher bond strength compared to PV (p < 0.0001). After 1-year water storage, the surfaces that were treated with SU_adhesive_ or with plasma+SU_adhesive_ showed a significant decrease in bond strength, except for the RU luting material that was used without any additional treatment. After aging, these three groups had the highest bond strengths, while PV used without treatment or with plasma produced lower bond strengths (p < 0.0001). When the same surface treatments and luting materials were used on Lava Esthetic, similar trends were observed. For this ceramic, after 24-h storage, RU used with SU_adhesive_ yielded significantly higher bond strengths, followed by PV with CP_primer_, plasma+ CP_primer_, and RU with plasma+ SU_adhesive_ or plasma alone, being statistically higher than untreated RU and untreated PV or PV with plasma (p < 0.0001). After 1 year, the luting material RU, whether used without treatment or treated with SU_adhesive_ or plasma+SU_adhesive_, was the only one able to mantain its bond strength to zirconia, ie, there was no statistically significant difference between 24 h and 1 year. The same effect was observed for Katana Zirconia. The combination of RU with SU_adhesive_ produced a significantly higher bond strength, followed by plasma+SU_adhesive_ and no treatment, which were all statistically significantly different from the other groups. Interestingly, RU without treatment was still significantly superior to RU with plasma or any treatment associated with PV. Within PV, CP_primer_ and plasma+ CP_primer_ produced higher bond strengths (p < 0.0001).

**Table 4:** Median (min – max) shear bond strength (MPa) for luting systems and ceramics at two evaluation times

Ceramic	Luting system		Time	
			24 h	1 year
Katana Zirconia	Panavia V5	No treatment	2.5 (1.6 – 3.5) Da	1.9 (0 – 4.3)**●** Cb
		Plasma	2.1 (1.9 – 3.5) Da	0 (0)✚ Db
		CP_primer_	16.2 (12.0 – 28.0) ABa	10.0 (9.0 – 17.6) Bb
		Plasma + CP_primer_	20.9 (19.4 – 28.4) Aa	9.8 (7.6 – 12.4) Bb
	RelyX Ultimate	No treatment	9.9 (7.2 – 17.1) Cb	12.4 (9.7 – 31.6) Aa
		Plasma	15.8 (11.8 – 21.2) Ba	9.4 (1.6 – 17.5) Bb
		SU_adhesive_	22.5 (16.7 – 26.2) Aa	19.1 (14.6 – 21.4) Aa
		Plasma + SU_adhesive_	18.7 (15.5 – 27.1) ABa	16.3 (7.9 – 21.5) Aa
Lava Esthetic	Panavia V5	No treatment	2.8 (1.7 – 4.3) Da	1.1 (0 – 2.7)**▲** Eb
		Plasma	1.9 (1.7 – 2.3) Da	0.7 (0 – 2.9)**■** Eb
		CP_primer_	18.2 (14.2 – 22.8) Ba	8.8 (7.4 – 11.3) CDb
		Plasma + CP_primer_	17.9 (10.1 – 23.3) Ba	7.5 (4.6 – 11.6) Db
	RelyX Ultimate	No treatment	11.6 (9.9 – 14.7) Ca	12.0 (9.3 – 21.5) Ba
		Plasma	15.1 (12.8 – 28.0) Ba	9.1 (3.0 – 21.6) Cb
		SU_adhesive_	23.6 (20.3 – 27.2) Aa	21.3 (14.5 – 25.9) Aa
		Plasma + SU_adhesive_	16.4 (13.9 – 27.9) Ba	14.0 (11.3 – 22.6) Ba

Medians (n = 8) followed by the same letter (uppercase compares rows [luting systems within each time]; lowercase compares columns [time within each luting system]) are not statistically significantly different (p>0.05). The different ceramics (Katana Zirconia and Lava Esthetic) were not compared. The following are the number of pre-test failures per group as identified by symbols: **✚**3 **●**8 **▲**3 **■**3. These values were included in the statistical analysis. CP_primer_: Clearfil Ceramic Primer; LC: light curing; SC: self-curing; SU_adhesive_: Scotchbond Universal.

**Fig 2:**
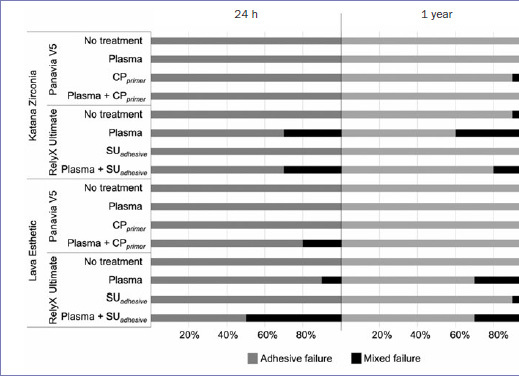
Distribution (%) of failure modes of the luting systems to zirconia after 24 h and after 1 year of storage in water.

The modes of failure are shown in Fig 2. Most of the specimens exhibited complete debonding of luting material from the zirconia. However, in 10% to 50% of the specimens tested at 24 h, the groups treated with plasma or plasma together with primers, showed mixed failures that involved both adhesive and cohesive failure within the luting material. In the specimens aged for 1 year, the percentage of mixed failures dropped to 10% to 40%, which was found in groups treated with CP_primer_ or SU_adhesive_, plasma alone, or a combination of both these treatments.

## DISCUSSION

The improvement in the polymerization of luting materials, when used in combination with the primers, was material-dependent. There was a significant increase in the DC of PV when the TP_primer_ was applied in both polymerization modes. There was also a faster polymerization rate when materials were light activated, ie, dual curing (Table 2). The percentage gain in conversion was greater in the self-curing mode (4.6% for self-curing compared to 1.9% for dual curing), along with a faster rate of increase in the degree of conversion (Fig 1). According to the manufacturer, TP_primer_ contains accelerators (Table 1) that are presumably responsible for these outcomes. In contrast, the addition of SU_adhesive_ did not affect polymerization kinetics or the final DC of RU. Thus, the first hypothesis, that the adhesive primers would increase the DC and polymerization rate of luting materials, was accepted for PV and rejected for RU. SU_adhesive_ contains silane and 10-MDP monomer, making it suitable as a ceramic surface-treatment primer. However, its initiator system is based on the conventional camphorquinone and amine combination (Table 1). Only one study was found in the literature^22^ that evaluated the effect of adhesives on the degree of conversion of luting materials. However, that study evaluated different resin-based luting systems than those examined in the present study, where no differences were observed for SU_adhesive_ used with RU or ED Primer and Panavia F. The different results for the versions of Panavia are probably due to differences in their compositions.

Regardless of primer application, as expected,^3,8,16,25^ both systems had a significantly lower polymerization rate and DC when used in self-curing mode. Despite the fact that similar values were reported for PR_max_ for both self-curing materials, the degree of conversion after 10 min was approximately 50% less for RU; but for PV, this decrease was only ~17% compared to the same material in dual-curing mode (Table 2). A lower DC may jeopardize the physicochemical properties and accelerate long-term degradation of the luting material.^7,28^ The microhardness test was performed to evaluate where these differences in polymerization might compromise the mechanical properties at the top surface closest to the light. The test showed that even after 24 h, when light cured with 17.2 J/cm^2^ of energy from the Valo curing light, these luting materials were harder than the self-cured luting materials (Table 3). Thus, the second hypothesis was accepted. Since the microhardness test evaluates properties at the surface, evaluations were not performed in combination with the application of the primers which would be present at this location, as they might interfere with measurements. Similar microhardness outcomes were reported for RU, while the polymerization modes had an effect on PV. A previous study reported significantly lower tensile strength for RU when it was allowed to self-cure.^13^ At the same time, another study^28^ reported no difference between polymerization modes for PV after 24 h. However, both materials showed lower mechanical properties after thermocycling when they were self-cured. The poor performance when the luting material was allowed to self-cure is relevant for light-compromised situations in which thick or opaque indirect materials are used,^5,11,23^ it is difficult to place the light-unit tip perpendicular and close to the restoration,^1^ and inhomogeneity exists in the irradiance or wavelength of light output.^26^

The bond strength of the resin-based luting materials to zirconia was improved when the primers were used (Table 4). Thus, the third hypothesis was accepted. Here, PV was used with a non-polymerizable CP_primer_ containing silane and 10-MDP; the latter includes both phosphate and reactive phosphonate groups that can chemically bond to zirconium oxide (P-O-Zr).^15^ Previous studies also reported increased bond strength for this system.^36^ In contrast, SU_adhesive_ is a dental adhesive that can also be used as a primer for ceramics because it contains both silane and 10-MDP. These universal adhesives have been reported to be a good alternative for treating the zirconia surface.^6^ After 24 h, no major differences were observed between luting systems. In contrast, after 1 year of storage in water, RU, when used in combination with SU_adhesive_, maintained a reasonably good bond to zirconia. However, the SBS of PV used in combination with CP_primer_ showed a significant reduction (39% reduction for Katana Zirconia and 51% for Lava Ultimate; Table 4). Other studies have also demonstrated a higher SBS to zirconia when SU_adhesive_ and RU were used,^24,38,40^ although most studies have reported a decrease in SBS after aging both resin-based luting systems for one year.^24,40^

Variations in the methodology of previous studies must be considered when interpreting the results. As demonstrated when evaluating the polymerization kinetics, PV achieved a higher conversion when used with TP_primer_. In the SBS test, only the adhesion to ceramic was measured and not to tooth substrate. Also, TP_primer_ was not used. This may have compromised the DC of PV and consequently affected the bond strength outcomes,^18,33^ especially when specimens were subjected to artificial aging, where a lower DC accelerates hydrolytic degradation. One study reported diminished bond strength to dentin when these same luting materials were not light activated but instead left to self-cure.^13^ Significantly lower SBS was observed for all PV groups regardless of surface treatment (Table 4). This may have been due to the absence of TP_primer_, which resulted in reduced polymerization of the resin (Table 2). One study evaluating the bond strength of zirconia to dentin using RU and SU_adhesive_ as well as the previous version, Panavia F system with CP_primer_, reported greater bond strengths for RU after 24 h. However, after aging, both adhesives performed similarly, with significantly lower bond strength.^40^ In this study, after 1-year water storage, pre-test failures were observed when CP_primer_ was not used (Table 4), which confirms the beneficial effect of the TP_primer_, even after aging.

It is noteworthy that most (> 50%) of the groups failed adhesively, ie, there was complete debonding of luting materials from the ceramic surface. When RU was used with SU_adhesive_ and/or plasma, some mixed failures were found (Fig 2). SU_adhesive_ is a combination of high and low molecular weight monomers resulting in a viscous liquid solution, but less viscous than the luting material. Thus, it can penetrate and wet the rough sandblasted surface and further improve the wetting of the luting material. This provides intimate contact between ceramic and luting material, which may favor better mechanical interlocking and possibly chemical bonding at the interface.^2^ In contrast, CP_primer_ is a liquid mixture of solvent and proprietary chemicals. Although it does not form a visible layer on the surface after solvent evaporation, it still influences wettability through chemical changes created at the surface to be be wet by the viscous luting material.^15^

The use of an alternative zirconia surface treatment was also addressed in this study. Zirconia has a low concentration of hydroxyl groups at the surface, and this produces a hydrophobic surface. Treatment with non-thermal atmospheric argon plasma can reduce carbon-based contaminants and increase the number of oxygen species, thus improving surface hydrophilicity.^14,37^ Cleaning and activating the surface raises wettability and surface energy, which favors resin bonding.^10^ A beneficial effect of plasma treatment was observed for RU compared to the untreated surfaces at 24 h; however, after 1 year, the opposite occurred (Table 4). The improved hydrophilicity appears to have contributed to the degradation of the interface. Other studies have also demonstrated a better immediate bond strength to zirconia after plasma treatment,^37^ but this benefit was lost after aging.^2^ The combination of plasma treatment with primers did not enhance SBS (Table 4), which corroborates previous studies.^17,20^ In general, surface treatment with non-thermal atmospheric plasma did not improve the adhesion of luting material to zirconia in this study. Thus, the fourth hypothesis was rejected. Although different types of zirconia may exhibit different behaviors according to their composition and atomic arrangement, no differences in SBS were found between the two zirconia materials used in this study (p = 0.7744).

This study design was limited in that the microhardness of the resin-based luting materials was evaluated only in the short term, to be compared with the degree of conversion data, but a long-term evaluation (1 year) would have been interesting to enable comparisons with bond strength data. In addition, future studies should compare the adhesion to zirconia after both CP_primer_ and TP_primer_ were used. Ideally, the bond to both tooth and zirconia should be evaluated simultaneously.

## CONCLUSION

1. The degree of conversion was only improved for PV when the TP primer was used. There was no effect on the polymerization kinetics of RU when SU_adhesive_ was used.
2. When used in self-cure mode, PV demonstrated better performance than RU. However, light curing was required to achieve a high degree of conversion for both resin-based luting materials.
3. Light curing the resin-based luting material produced a greater microhardness of both materials within the first 24 h.
4. For both luting systems, the bond strength to zirconia ceramics improved when the adhesive primer was used. After 1 year of water storage, only RU, when used with SU_adhesive_, maintained its bond strength to zirconia. There was a significant reduction in the bond strength of PV to Katana Zircona (39% reduction) and to Lava Ultimate (51% reduction) after one year.

## Clinical relevance:

Light curing of dual-cure resin-based luting materials is recommended. The use of primers/adhesives in luting systems is recommended to improve bonding to zirconia ceramics. Panavia V5 (Kuraray Noritake) used with Tooth Primer increased the luting material polymerization. Only RelyX Ultimate (3M Oral Care) used with Scotchbond Universal maintained bond strengths to zirconia after 1 year of storage.
